# Serum alanine aminotransferase/aspartate aminotransferase ratio is one of the best markers of insulin resistance in the Chinese population

**DOI:** 10.1186/s12986-017-0219-x

**Published:** 2017-10-10

**Authors:** Li Zhao, Jing Cheng, Yingchao Chen, Qin Li, Bing Han, Yi Chen, Fangzhen Xia, Chi Chen, Dongping Lin, Xuemei Yu, Ningjian Wang, Yingli Lu

**Affiliations:** 1 Institute and Department of Endocrinology and Metabolism, Shanghai Ninth People’s Hospital, Shanghai JiaoTong University School of Medicine, Shanghai, 200011 China; 2 Department of Endocrinology and Metabolism, Shanghai Fengxian District Central, Hospital, Shanghai, 201400 China

**Keywords:** Alanine aminotransferase/aspartate aminotransferase ratio, Insulin resistance, Non-alcoholic fatty liver disease

## Abstract

**Background:**

The alanine aminotransferase (ALT)/aspartate aminotransferase (AST) ratio is reportedly associated with insulin resistance (IR). However, few studies have explored the relationship between the ALT/AST ratio and IR in the Chinese population. Here, we aimed to explore whether the ALT/AST ratio is associated and, if so, to what extent, with IR in the Chinese population as categorized by waist circumference (WC).

**Methods:**

Our data were obtained from the SPECT-China study, a cross-sectional survey on the prevalence of metabolic diseases and risk factors in East China from 2014 to 2015. A total of 8398 participants aged 52.16 ± 13.16 (mean ± standard deviation) years were included in this study. Anthropometric indices, biochemical parameters and clinical characteristics were measured. IR was defined as the top quartile of the homeostasis model assessment of insulin resistance (HOMA-IR > 1.6), and central obesity was defined as a WC ≥90 cm in males or ≥80 cm in females. Linear regression and receiver operating characteristic curve analyses were conducted.

**Results:**

The ALT/AST ratio was significantly correlated and associated with HOMA-IR in both non-centrally obese (*r* = 0.284, B = 0.509, 95% confidence interval (CI): 0.459–0.559, *P* < 0.001) and centrally obese subjects (*r* = 0.372, B = 0.607, 95%CI: 0.532–0.683, *P* < 0.001) after adjusting for potential confounders. The ALT/AST ratio was one of the best markers of IR, with areas under the curve (AUC) values of 0.66 (0.64–0.68) in non-centrally and 0.68 (0.66–0.70) in centrally obese subjects. In the prediction model for IR, the AUCs were significantly augmented after adding the ALT/AST ratio in both non-centrally obese [AUC 95%CI 0.69(0.67–0.71) vs 0.72(0.70–0.74), *P*<0.001] and central obese [AUC 95%CI 0.69(0.67–0.71) vs 0.73(0.72–0.75), *P*<0.001] subjects. The optimal cut-off points of the ALT/AST ratio for identifying IR were 0.80 in non-centrally obese people and 0.78 in centrally obese people, respectively.

**Conclusion:**

The ALT/AST ratio may be one of the best markers for IR in the Chinese population. Whether the ALT/AST ratio should be regarded as an additional metabolic syndrome component in the Chinese population warrants further investigation.

**Electronic supplementary material:**

The online version of this article (10.1186/s12986-017-0219-x) contains supplementary material, which is available to authorized users.

## Background

Obesity is an increasingly common problem worldwide [1] and is a major determinant of insulin resistance (IR) [2]. IR is a risk factor for the incidence of a number of chronic diseases, such as hypertension, dyslipidemia, type 2 diabetes mellitus (T2DM) and cardiovascular disease(CVD) [3]. At present, accurate measurement of IR is acquired by using the glucose clamp technique, which, due to its time and expense, is almost impossible to perform in large epidemiological studies. Thus, simple alternatives are sought, including the homeostatic model assessment of insulin resistance (HOMA-IR), which has been widely used in large population-based samples [4] and correlates well with clamp results [5]. However, HOMA-IR is calculated based on fasting insulin and glucose levels [5]. Insulin levels are not measured in routine health examinations and clinical practice, which makes the use of this index limited. Thus, it is necessary to find a more common, reliable and simple indicator to identify IR.

Non-alcoholic fatty liver disease (NAFLD) is considered the hepatic manifestation of metabolic syndrome (MS), and excess adiposity and IR represent its two major risk factors [6]. Elevated liver enzymes including alanine aminotransferase (ALT), aspartate aminotransferase (AST) and gamma-glutamyl transpeptidase (GGT) as well as the ALT/AST ratio are commonly used as surrogate markers of NAFLD [7–9]. Many studies have revealed a positive correlation between elevated liver enzyme levels and obesity-related diseases including T2DM [10, 11], MS [8, 12–14], and CVD [10, 15]. These levels have also been demonstrated to be associated with indirect measurements of IR, including fasting insulin levels [12] and HOMA-IR [11]. However, few studies have reported an association between the ALT/AST ratio and IR [15], and no studies have investigated the relationship between the ALT/AST ratio and IR in the general Chinese adult population. Therefore, the specific objective of this study was to investigate whether and to what extent the ALT/AST ratio was associated with insulin resistance in the general Chinese population as categorized by waist circumference (WC).

## Methods

### Study population

**Fig. 1:**
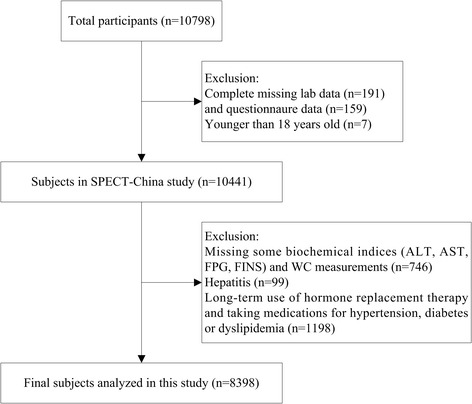
Flowchart of the participants selected from SPECT-China

SPECT-China is a cross-sectional survey of the prevalence of metabolic diseases and risk factors in East China (ChiCTR-ECS-14,005,052, www.chictr.org.cn). A stratified cluster sampling method was used. The first sampling level was by rural and urban residence, and the second was by area economic status. From February 2014 to December 2015, a total of 22 sites in Shanghai, Jiangxi Province, Zhejiang Province, Jiangsu Province and Anhui Province were selected. Adults aged 18 years and older who were Chinese citizens and had lived in their current residence for 6 months or longer were included. Those with severe communication problems or acute illness or who were unwilling to participate were excluded from the study. A total of 10,798 residents participated in this investigation. After excluding participants with missing laboratory results (without any lab data, *n* = 191) or questionnaire data (*n* = 159) and who were younger than 18 years (*n* = 7), 10,441 subjects were enrolled in the SPECT-China study. Participants (*n* = 746) with missing part laboratory values (ALT, AST, FPG, fasting insulin) and anthropometric data were also excluded. Participants with viral hepatitis (*n* = 99) or who took taking medications (*n* = 1198) for hypertension, diabetes or dyslipidemia were excluded. Finally, 8398 subjects with a mean ± SD age of 52.16 ± 13.16 years were included in this study (Fig. 1). The study protocol was approved by the Ethics Committee of Shanghai Ninth People’s Hospital, Shanghai JiaoTong University School of Medicine. All procedures were performed in accordance with the ethical standards of the committees responsible for human experimentation (institutional and national) and with the Helsinki Declaration of 1975, as revised in 2008. All participants provided written informed consent before data collection.

### Measurements

At every site, the same trained staff group completed the questionnaire by including information on demographic characteristics, medical history and lifestyle risk factors [16]. Current smoking was defined as having smoked at least 100 cigarettes in one’s lifetime or currently smoking cigarettes and current drinking was defined as alcohol intake more than once per month during the past 12 months [16]. Body weight, height, WC and blood pressure were measured using standard methods as described previously [16]. Body mass index (BMI) was calculated as the body weight in kilograms divided by the height in meters squared. The waist-to-hip ratio (WHR) was calculated as the WC divided by the hip circumference (HC).

Venous blood samples were drawn after an overnight fast of at least 8 h. The blood samples for plasma glucose test were collected into vacuum tubes containing the anticoagulant sodium fluoride and were centrifuged on location within 1 h after collection. Blood samples were stored at −20 °C and shipped by air in dry ice to a central laboratory (certified by the College of American Pathologists) within 2–4 h. Glycated hemoglobin (HbA1c) was assessed by high-performance liquid chromatography (MQ-2000PT, China). Fasting plasma glucose (FPG), ALT, AST, total cholesterol (TC), triglycerides (TG), high-density lipoprotein (HDL) and high-density lipoprotein (LDL) were measured using BECKMAN COULTER AU 680 (Germany). Fasting insulin (FINS) was measured using the chemiluminescence method (Abbott i2000 SR, USA). IR was estimated by calculating the HOMA-IR index as follows: [FINS (pmol/L) × FPG (mg/dl)]/(22.5 × 6.965).

### Definition of variables and outcomes

Insulin resistance was defined according to the top quartile of HOMA-IR in our study (HOMA-IR >1.6). In the sensitivity analysis, insulin resistance was defined as a HOMA-IR score >2 [17]. Central obesity was defined as a WC ≥90 cm in males or ≥80 cm in females [16]. We used residence area as a covariate because in China, the prevalence of obesity in rural and urban areas may differ [18]. Economic status was estimated based on the mean gross domestic product per capita of the whole nation (6807 US dollars according to the World Bank) in 2013 as the cutoff point for each site.

### Statistical analysis

We analyzed the survey results using IBM SPSS Statistics, Version 22 (IBM Corporation, Armonk, NY, USA). Continuous variables that fitted a Gaussian distribution and categorical variables were expressed as the means ± standard deviation (SD) and as percentages (%), respectively. Continuous variables with a skewed distribution were presented as medians (interquartile range) and were log-transformed for analysis. To test the differences between participants with and without central obesity, the Mann-Whitney U and Student t tests were used for non-normally and normally distributed continuous data respectively, and the Pearson χ2 test was used for categorical variables. Spearman’s correlation coefficient was employed to test the correlations between potential metabolic factors and HOMA-IR. The association of the ALT/AST ratio (independent variable) with HOMA-IR (dependent variable) was assessed by linear regression. Model 1 did not adjust for any factor. Model 2 adjusted for age, gender, smoking, drinking, residence area, economic status, BMI, SBP, HbA1c and TG. Binary logistic regression analysis was also conducted to explore the association of the ALT/AST ratio and other possible metabolic factors with IR. Finally, receiver operating characteristic (ROC) curve analyses were conducted to determine optimal ALT/AST ratio cutoff points for insulin resistance in both non-central and central obesity. Areas under ROC curves (AUC) were calculated for each potential metabolic factor to identify which factor was a better predictor of IR. The Z test was performed to examine differences between AUCs. Data were expressed as the AUC (95% confidence interval (CI)). Because HOMA-IR was calculated by an equation including FPG and insulin as terms, FPG was not included in the ROC curve analysis. A two-sided *P* value <0.05 was considered significant.

## Results

### Characteristics of the study population

The general characteristics of the participants were summarized in Table 1, as categorized by WC. Among the 8398 subjects, there were 3414 (40.7%) men aged 53.04 ± 13.46 years and 4984 (59.3%) women aged 51.57 ± 12.91 years. The mean WC was 79.63 ± 10.16 cm, and 30.9% (2795/5603) of the subjects exhibited central obesity. Among the participants, the prevalence of IR was 8.7% in the non-centrally obese subjects and 29.9% in those with central obesity. Compared with non-centrally obese subjects, subjects with central obesity were older and had significantly greater BMI and blood pressure, as well as greater levels of HOMA-IR, ALT, AST and the ALT/AST ratio. In contrast, the level of HDL was significantly lower in this group. In addition, the differences between these two groups in terms of residence area, economic status, smoking and alcohol consumption were also statistically significant (all *P* < 0.001).

**Table 1:** General characteristics of the study participants

Characteristics	Total	Non-central obesity	Central obesity	*P*
N	8398	5603	2795	
Male (%)	40.7	45.5	30.9	<0.001
Age (y)	52.16 ± 13.16	49.91 ± 13.23	56.69 ± 11.76	<0.001
Weight (kg)	63.26 ± 11.10	60.01 ± 9.44	69.80 ± 11.31	<0.001
WC (cm)	79.63 ± 10.16	74.67 ± 7.32	89.58 ± 7.36	<0.001
HC (cm)	93.00 ± 6.93	90.43 ± 5.45	98.17 ± 6.71	<0.001
BMI (kg/m^2^)	24.27 ± 3.50	22.87 ± 2.70	27.08 ± 3.22	<0.001
WHR	0.86 ± 0.09	0.83 ± 0.07	0.92 ± 0.10	<0.001
SBP (mmHg)	130.36 ± 21.48	126.27 ± 20.32	138.53 ± 21.41	<0.001
DBP (mmHg)	78.74 ± 13.09	76.67 ± 12.57	82.86 ± 13.15	<0.001
FPG (mmol/L)	5.27 (4.90,5.73)	5.20 (4.86,5.61)	5.40 (4.99,6.00)	<0.001
HbA1c (%)	5.43 ± 0.83	5.31 ± 0.74	5.67 ± 0.95	<0.001
FINS (pmol/L)	32.40 (22.80,46.30)	28.70 (20.60,40.20)	41.70 (30.20,58.40)	<0.001
HOMA-IR	1.11 (0.76,1.63)	0.97 (0.68,1.37)	1.47 (1.03,2.16)	<0.001
IR (%)	15.8	8.7	29.9	<0.001
TG (mmol/L)	1.27 (0.92,1.85)	1.14 (0.85,1.63)	1.58 (1.16,2.22)	<0.001
TC (mmol/L)	5.14 ± 1.02	5.01 ± 0.98	5.39 ± 1.04	<0.001
LDL (mmol/L)	3.07 ± 0.78	2.95 ± 0.74	3.32 ± 0.80	<0.001
HDL (mmol/L)	1.45 ± 0.31	1.49 ± 0.32	1.39 ± 0.29	<0.001
ALT (U/L)	18 (13,25)	17 (13,23)	19 (15,29)	<0.001
AST (U/L)	23 (19,28)	22 (19,27)	24 (20,29)	<0.001
ALT/AST	0.77 (0.63,0.97)	0.75 (0.61,0.94)	0.83 (0.67,1.05)	<0.001
Rural residence (%)	61.1	57.9	67.3	<0.001
Economic status (high,%)	58.4	60.4	54.5	<0.001
Current smoker (%)	21.3	22.9	18	<0.001
Alcohol consumption (%)	12.6	12	13.6	0.038

Continuous and categorical variables were expressed as the means ± standard deviation (SD) and percentages (%), respectively. Data for FPG, FINS, HOMA-IR, TG, ALT, AST and ALT/AST were skewed and were presented as medians (interquartile range). The Mann-Whitney U test and the Student t test were used for non-normally and normally distributed continuous variables, respectively, and the Pearson χ2 test was used for categorical variables

*WC* waist circumference, *HC* hip circumference, *BMI* body mass index, *WHR* waist to hip ratio, *SBP* systolic blood pressure, *DBP* diastolic blood pressure, *FPG* fasting plasma glucose, *HbA1c* glycated hemoglobin, *FINS* fasting insulin, *HOMA-IR* homeostasis model assessment-insulin resistance, *IR* insulin resistance, *TG* triglycerides, *TC* total cholesterol, *LDL* low-density lipoprotein, *HDL* high-density lipoprotein, *ALT* alanine aminotransferase, *AST* aspartate aminotransferase

### Correlation between the ALT/AST ratio and HOMA-IR

Table 2 showed that both in subjects with and without central obesity, HOMA-IR was significantly and positively correlated with WC, BMI, blood pressure, TG, ALT, and the ALT/AST ratio and was negatively correlated with HDL. We also observed that among the measured parameters, the ALT/AST ratio was the marker that best correlated with HOMA-IR both in non-centrally obese (*r* = 0.284, *P* < 0.001) and centrally obese subjects (*r* = 0.372, *P* < 0.001). To further investigate whether the ALT/AST ratio can explain the changes in HOMA-IR levels independently of other known confounding factors, linear regression analysis was applied. The results showed that the ALT/AST ratio was independently and significantly associated with HOMA-IR in both non-centrally obese (B = 0.509, 95% CI 0.459–0.559, *P* < 0.001) and centrally obese subjects (B = 0.607, 95% CI 0.532–0.683, *P* < 0.001) after adjusting for age, gender, smoking, drinking, residence area, economic status, BMI, SBP, HbA1c and TG. When Ln ALT/AST ratio increased 0.1 unit increment, B values were 0.056 (95% CI 0.051–0.060) in total subjects, 0.051 (95% CI 0.046–0.056) in subjects without central obesity and 0.061 (95% CI 0.053–0.068) in subjects with central obesity, respectively, about 10 times smaller than those of every 1 unit increment (Table 3). All these implied the ALT/AST ratio was significantly correlated with HOMA-IR.

**Table 2:** Correlation between HOMA-IR and measured parameters

Potential	Total		Non-central obesity		Central obesity	
metabolic factors	r	*P*	r	*P*	r	*P*
WC (cm)	0.329	<0.001	0.15	<0.001	0.169	<0.001
BMI (kg/m^2^)	0.404	<0.001	0.266	<0.001	0.302	<0.001
SBP (mmHg)	0.134	<0.001	0.047	<0.001	0.05	0.008
DBP (mmHg)	0.128	<0.001	0.047	0.001	0.078	<0.001
HbA1c (%)	0.131	<0.001	0.004	0.785	0.187	<0.001
TG (mmol/L)	0.327	<0.001	0.235	<0.001	0.294	<0.001
TC (mmol/L)	0.081	<0.001	0.013	0.33	0.027	0.148
LDL (mmol/L)	0.118	<0.001	0.053	<0.001	0.015	0.423
HDL (mmol/L)	−0.239	<0.001	−0.213	<0.001	−0.186	<0.001
ALT (U/L)	0.216	<0.001	0.125	<0.001	0.27	<0.001
AST (U/L)	−0.049	<0.001	−0.133	<0.001	0.013	0.499
ALT/AST	0.343	<0.001	0.284	<0.001	0.372	<0.001

*WC* waist circumference, *BMI* body mass index, *SBP* systolic blood pressure, *DBP* diastolic blood pressure, *HbA1c* glycated hemoglobin, *HOMA-IR* homeostasis model assessment-insulin resistance, *TG* triglycerides, *TC* total cholesterol, *LDL* low-density lipoprotein, *HDL* high-density lipoprotein, *ALT* alanine aminotransferase, *AST* aspartate aminotransferase. The data were Spearman’s correlation coefficients

**Table 3:** Linear regression analysis for the correlation between the ALT/AST ratio and HOMA-IR

	Total		Non-central obesity		Central obesity	
	Model 1	Model 2	Model 1	Model 2	Model 1	Model 2
B(per 1 unit)	0.668	0.555	0.548	0.509	0.685	0.607
95%CI	0.628–0.708	0.513–0.597	0.501–0.595	0.459–0.559	0.617–0.753	0.532–0.683
R^2^	0.114	0.269	0.085	0.195	0.123	0.240
*P*	<0.001	<0.001	<0.001	<0.001	<0.001	<0.001
B(per 0.1 unit)	0.067	0.056	0.055	0.051	0.069	0.061
95%CI	0.063–0.071	0.051–0.060	0.050–0.060	0.046–0.056	0.062–0.075	0.053–0.068
R^2^	0.115	0.269	0.086	0.196	0.123	0.240
*P*	<0.001	<0.001	<0.001	<0.001	<0.001	<0.001

Because the ALT/AST ratio and HOMA-IR were non-normally distributed, the values were Ln transformed for analysis. Model 1 did not adjust for any factor. Model 2 adjusted for age, gender, smoking, drinking, residence area, economic status, BMI, SBP, HbA1c and TG

### Association of the ALT/AST ratio and other metabolic factors with insulin resistance

In our study, the top quartile of HOMA-IR is 1.6. When IR is defined as HOMA-IR >1.6, logistic regression analyses showed that the ALT/AST ratio remained strongly associated with insulin resistance after adjusting for the potential confounding factors. The odds ratios (OR) for insulin resistance were 1.90 (1.74–2.06) in non-central obesity and 2.06 (1.85–2.28) in central obesity (Table 4).

**Table 4:** Associations of IR (HOMA-IR > 1.6 or 2) with ALT/AST and other metabolic factors in logistic regression analyses

	HOMA-IR>1.6		HOMA-IR>2	
	Non-central obesity	Central obesity	Non-central obesity	Central obesity
ALT/AST	1.90(1.74–2.06)***	2.06(1.85–2.28)***	1.89(1.70–2.11)***	2.02(1.81–2.25)***
ALT	1.55(1.44–1.68)***	1.58(1.44–1.72)***	1.46(1.33–1.61)***	1.64(1.49–1.80)***
AST	1.08(1.00–1.17)	1.07(0.99–1.16)	0.98(0.88–1.09)	1.17(1.08–1.28) ***
TG	1.72(1.59–1.85)***	1.60(1.46–1.75)***	1.67(1.52–1.83)***	1.63(1.48–1.79)***
HDL	0.64(0.60–0.70)***	0.71(0.65–0.78)***	0.63(0.57–0.70) ***	0.69(0.62–0.76)***
BMI	2.02(1.83–2.23)***	1.87(1.67–2.09)***	1.93(1.70–2.18) ***	1.89(1.67–2.13)***
SBP	1.26(1.16–1.38)***	1.19(1.09–1.31)***	1.28(1.14–1.44) ***	1.24(1.12–1.37)***
DBP	1.18(1.09–1.28)***	1.16(1.07–1.27) **	1.13(1.01–1.25) *	1.20(1.09–1.32) ***

Data were odds ratio (95% confidence interval). *HOMA-IR* homeostasis model assessment-insulin resistance, *ALT* alanine aminotransferase, *AST* aspartate aminotransferase, *TG* triglycerides, *HDL* high-density lipoprotein, *BMI* body mass index, *WC* waist circumference, *SBP* systolic blood pressure, *DBP* diastolic blood pressure

Adjusted odds ratios for each 1-SD increment of each potential risk factor associated with insulin resistance were calculated. The model has been adjusted for age, gender, smoking, drinking, residence area, economic status and HbA1c. *P<0.05; **P<0.01; ***P<0.001

### Areas under ROC curves for potential metabolic markers of insulin resistance

ROC curve analyses revealed that the ALT/AST ratio was one of the best markers of IR, with an area under the ROC curve of 0.66 (0.64–0.68) in non-centrally obese and 0.68 (0.66–0.70) in centrally obese subjects. The optimal cutoff points of the ALT/AST ratio for identifying IR were 0.80 in non-centrally obese and 0.78 in centrally obese subjects. In the non-centrally obese subjects, the ALT/AST ratio had comparable power to identify IR as those of BMI [AUC 0.64(0.63–0.66); vs AUC of the ALT/AST ratio, *P* = 0.22] and TG [AUC 0.64(0.63–0.66); vs AUC of the ALT/AST ratio, *P* = 0.21], while in the centrally obese subjects, the ALT/AST ratio had the greatest power. However, the ALT/AST ratio was better than other traditional MS components, including blood pressure and HDL, at predicting IR. Besides, we established two prediction models for IR. Model 1 included age, gender, BMI, SBP, TG, LDL and HDL. Model 2 further included ALT/AST. The results showed that the AUCs were significantly augmented, when adding ALT/AST into the prediction model in both non-centrally obese (AUC 95% CI 0.69(0.67–0.71) of Model 1 vs 0.72(0.70–0.74) of Model 2, *P*<0.001) and central obese (AUC 95% CI 0.69(0.67–0.71) of Model 1 vs 0.73(0.72–0.75) of Model 2, *P*<0.001) subjects. This implied that the ALT/AST ratio had a great power to identify IR (Table 5).

**Table 5:** AUC(95%CI) of markers for insulin resistance (HOMA-IR>1.6) in subjects categorized by WC

	Non-central obesity			Central obesity		
	AUC(95%CI)	*P*1	*P*2	AUC(95%CI)	*P*1	*P*2
ALT/AST	0.66(0.64–0.68)	<0.001		0.68(0.66–0.70)	<0.001	
ALT	0.60(0.58–0.62)	<0.001	<0.001	0.64(0.62–0.66)	<0.001	<0.001
AST	0.48(0.46–0.50)	0.016	<0.001	0.52(0.49–0.54)	0.191	<0.001
TG	0.64(0.63–0.66)	<0.001	0.21	0.64(0.62–0.66)	<0.001	0.002
HDL	0.39(0.37–0.41)	<0.001	0.002	0.41(0.39–0.43)	<0.001	<0.001
BMI	0.64(0.63–0.66)	<0.001	0.22	0.65(0.63–0.67)	<0.001	0.006
WC	0.60(0.58–0.62)	<0.001	<0.001	0.60(0.58–0.62)	<0.001	<0.001
SBP	0.53(0.51–0.55)	0.001	<0.001	0.53(0.51–0.55)	0.007	<0.001
DBP	0.53(0.51–0.55)	0.001	<0.001	0.55(0.52–0.57)	<0.001	<0.001
Model 1	0.69(0.67–0.71)	<0.001		0.69(0.67–0.71)	<0.001	
Model 2	0.72(0.70–0.74)^*^	<0.001		0.73(0.72–0.75)^*^	<0.001	

Data were expressed as area under curve (95% confidence interval). Model 1 included age, gender, BMI, SBP, TG, LDL and HDL. Model 2 further included ALT/AST. *ROC* receiver operating characteristics, *AUC* area under ROC curve, *HOMA-IR* homeostasis model assessment-insulin resistance, *ALT* alanine aminotransferase, *AST* aspartate aminotransferase, *TG* triglycerides, *HDL* high-density lipoprotein, *BMI* body mass index, *WC* waist circumference, *SBP* systolic blood pressure, *DBP* diastolic blood pressure. P1: The diagnostic value for ROC, two tail significance. P2: Difference of AUC compared to the ALT/AST ratio model, two tail significance (Z test). ^*^: *P*<0.001, difference of AUCs between Model 1 and Model 2, two-tailed significance (Z test)

### Sensitivity analyses

When insulin resistance is defined as HOMA-IR >2, the ALT/AST ratio remained one of the best markers for IR in the Chinese population with OR 1.89 (1.70–2.11) in non-central obesity and 2.02 (1.81–2.25) in central obesity (all *P* < 0.001) after adjusting for all possible confounders (Table 4). Moreover, ROC curve analyses revealed that the ALT/AST ratio also had comparable power to identify IR to that of BMI and TG in non-centrally obese subjects and had comparable power with BMI in centrally obese subjects. In the two prediction models for IR, the ALT/AST ratio also showed greater power to evaluate IR (Additional file 1: Table S1). Besides, to comprehensively explore the relationship between the ALT/AST ratio and IR, we further divided the subjects into four groups (non-obese normal WC group, non-obese centrally obese group, obese normal WC group and obese centrally obese group), according BMI and WC. Linear regression analysis displayed that the ALT/AST ratio was also independently and significantly correlated with HOMA-IR. After controlling the confounding factors, the β values of the ALT/AST ratio were the highest, which were 0.29, 0.26, 0.32 and 0.35, respectively, in the four groups (Additional file 1: Table S2). Logistic regression analysis also showed the strong association of the ALT/AST ratio with HOMA-IR. The ORs were 1.79 (1.57–2.05), 1.82 (1.41–2.36), 1.72 (1.41–2.08) and 2.00 (1.77–2.27), respectively. All these indicated that the ALT/AST ratio was one of the best markers for insulin resistance (Additional file 1: Table S3).

## Discussion

In this population-based study, we found that the ALT/AST ratio might be one of the best markers for insulin resistance in the Chinese population. The optimal cut-off point of the ALT/AST ratio to predict insulin resistance was 0.80 in non-obese and 0.78 in centrally obese subjects. After adjustment for potential confounders, the ALT/AST ratio remained significantly associated with insulin resistance in both non-centrally and centrally obese subjects. Comparison of the AUCs of the two prediction models for insulin resistance displayed the ALT/AST ratio had a great power to identify insulin resistance. Based on these findings, we postulated a hypothesis that the ALT/AST ratio might be included in research studies as an additional MS component for the Chinese population.

Although HOMA-IR has proved to be a robust tool for the assessment of IR, there still is no criterion by which a person can be identified as being IR [15]. According to the World Health Organization (WHO) [5], IR is usually defined as a value greater than the 75th percentile value of parameters for non-diabetic subjects. However, the cut-off points reported in the literature continue to vary widely [19–22]. We chose 2 as the HOMA-IR cut-off point because this cut-off point was found in the general healthy population [17, 23] and increased cardiovascular risk in the general population independent of traditional risk factors [24, 25]. Blood pressure, TG, HDL and obesity are traditional MS components and risk factors of IR. Our results showed that the ALT/AST ratio had a larger area under the curve for predicting IR than blood pressure, TG and WC in both non-centrally and centrally obese subjects. The ALT/AST ratio was also better associated with IR than ALT or AST alone. We also conducted a sensitivity analysis by using a HOMA-IR score >2. ROC curve analyses revealed that the ALT/AST ratio also had comparable power to identify IR to that of BMI and TG in non-centrally obese subjects and had comparable power with BMI in centrally obese subjects. In addition, when adding the ALT/AST ratio into the prediction model for IR, the predictive power was strengthen greatly, which manifested the ALT/AST ratio had a great influence on insulin resistance. As liver function is a low-cost and routine clinical measurement, use of the ALT/AST ratio is highly cost-effective and may have a promising future as a MS component in the Chinese population.

Several lines of evidence have shown the mechanism underlying the connection between the ALT/AST ratio and insulin resistance. NAFLD is closely associated with insulin resistance. Because adding NAFLD to ATP III criteria significantly improved their diagnostic accuracy for IR, Giovanni Musso suggested that NAFLD should be included in the definition of MS [17]. In an animal NAFLD model, even without changes in weight and muscle insulin resistance, the ability of insulin to suppress hepatic glucose production was diminished [26]. NAFLD or liver fatty infiltration may induce hepatic insulin resistance by activating PKC-epsilon and JNK1, which may interfere with the tyrosine phosphorylation of IRS-1 and IRS-2. Consequently, the ability of insulin to activate glycogen synthesis and inhibit gluconeogenesis is impaired [27]. It is also well known that ALT and the numerator of the ALT/AST ratio are closely related to liver fat accumulation and are surrogate markers for NAFLD [28]. Moreover, elevated levels of liver enzymes are positively associated with MS components [8, 12–14], and higher ALT levels and ALT/AST ratios have been found to be independently associated with IR [8]. In our linear regression analyses, even after adjusting for potential confounders including MS components, the ALT/AST ratio remained associated with IR; therefore, this association seems to be independent of MS, and the reason for this warrants further investigation.

A previous study also found that the ALT/AST ratio was the best surrogate marker for IR in non-obese Japanese adults [8]. Their subjects were categorized by BMI. In Chinese adults, WC may be a better alternative measure of fat distribution for predicting diabetic and cardiovascular risks than BMI [29] because abdominal/central obesity and visceral fat quantity predict disease risk more accurately [1]. In our subjects, Spearman’s correlation coefficient between WC and BMI was 0.74 (*P* < 0.001); therefore, WC was adopted as a stratification factor instead of BMI. Second, in the Japanese study, BMI and blood pressure were not included in the ROC curve analyses, and in multiple linear regression analyses, the β coefficient of the ALT/AST ratio was smaller than that of BMI and TG; therefore, declaring that the “ALT/AST ratio is the best surrogate marker for IR” might be far-fetched. In contrast, in our study, the power of the ALT/AST ratio to predict IR was compared with that of MS components including blood pressure, TG, HDL and BMI. FPG was not included because it is included in the equation calculating HOMA-IR.

Our study had several strengths. First, the current study is the first to find that the ALT/AST ratio may be an optimal marker of IR in the Chinese population, not only better than ALT or AST alone but also better than MS components. Second, this study was subject to strong quality control; the anthropometric measurements and questionnaires were all completed by the same trained research group, and the biomedical measurements were performed in the same laboratory, which was certified by the College of American Pathologists. Third, our study used a community-dwelling population-based design with a large sample size and including possible information on confounders. The results are more representative than that would be obtained in a clinic-based population.

However, our study also has some limitations. First, due to the cross-sectional nature of the study, we cannot draw a causal relationship between the ALT/AST ratio and IR. Second, this study recruited primarily Han Chinese, and the data may not be generalizable to other ethnic groups. Third, although we excluded subjects with self-reported viral hepatitis, we could not completely exclude other types of hepatitis among the subjects. We did not test for the hepatitis virus in this population, which may have influenced our results. Finally, gamma-glutamyltransferase (GGT) is also a commonly used marker for NAFLD [9]. However, we did not measure GGT in this population. In a future study, we would also measure this indicator.

## Conclusions

The ALT/AST ratio might be a better biomarker of IR than ALT, AST, blood pressure, lipid profile and adiposity in the Chinese population. The question of whether the ALT/AST ratio can be regarded as an additional MS component in the Chinese population warrants further investigation. Because the measurement of liver markers is inexpensive and routinely performed in clinical settings, these findings may have important clinical and public health implications.

## Additional file

Additional file 1: Table S1. AUC(95%CI) of markers for insulin resistance (HOMA-IR>2) in subjects categorized by WC. **Table S2.** Linear regression analysis for the correlation of ALT/AST ratio and other potential metabolic factors with HOMA-IR. **Table S3.** Associations of insulin resistance (HOMA-IR >2) with ALT/AST ratio and other potential metabolic factors in logistic regression analyses. (DOCX 20 kb)

## Abbreviations

ALT: Alanine aminotransferase
AST: Aspartate aminotransferase
BMI: Body mass index
DBP: Diastolic blood pressure
FINS: Fasting insulin
FPG: Fasting plasma glucose
GGT: Gamma-glutamyl transpeptidase
HbA1c: Glycated hemoglobin
HC: Hip circumference
HDL: High-density lipoprotein
HOMA-IR: Insulin resistance index
LDL: Low-density lipoprotein
MS: Metabolic syndrome
NAFLD: Non-alcoholic fatty liver disease
SBP: Systolic blood pressure
TC: Total cholesterol
TG: Triglycerides
WC: Waist circumference
WHR: Waist-to-hip ratio
