# Supplementary material for: Serum alanine aminotransferase/aspartate aminotransferase ratio is one of the best markers of insulin resistance in the Chinese population
Source: Nutr Metab (Lond). 2017 Oct 10;14:64. doi: 10.1186/s12986-017-0219-x (PMC5633891; doi:10.1186/s12986-017-0219-x)
Supplement: Additional file 1: Table S1. — AUC(95%CI) of markers for insulin resistance (HOMA-IR>2) in subjects categorized by WC. Table S2. Linear regression analysis for the correlation of ALT/AST ratio and other potential metabolic factors with HOMA-IR. Table S3. Associations of insulin resistance (HOMA-IR >2) with ALT/AST ratio and other potential metabolic factors in logistic regression analyses. (DOCX 20 kb) [file 12986_2017_219_MOESM1_ESM.docx]

**Table S1 AUC (95%CI) of markers for insulin resistance (HOMA-IR＞2) in subjects categorized by WC**

|  | Non-central obesity | | |  | Central obesity | | |
| --- | --- | --- | --- | --- | --- | --- | --- |
|  | AUC (95%CI) | *P*1 | *P*2 |  | AUC (95%CI) | *P*1 | *P*2 |
| ALT/AST | 0.66 (0.64-0.69) | ＜0.001 |  |  | 0.68 (0.66-0.70) | ＜0.001 |  |
| ALT | 0.59 (0.56-0.62) | ＜0.001 | ＜0.001 |  | 0.65 (0.62-0.67) | ＜0.001 | ＜0.001 |
| AST | 0.44 (0.42-0.48) | ＜0.001 | ＜0.001 |  | 0.54 (0.51-0.56) | ＜0.01 | ＜0.001 |
| TG | 0.65 (0.62-0.68) | ＜0.001 | 0.40 |  | 0.65 (0.63-0.67) | ＜0.001 | ＜0.05 |
| HDL | 0.38 (0.35-0.40) | ＜0.001 | ＜0.01 |  | 0.39 (0.37-0.42) | ＜0.001 | ＜0.001 |
| BMI | 0.63 (0.61-0.66) | ＜0.001 | 0.08 |  | 0.65 (0.63-0.68) | ＜0.001 | 0.05 |
| WC | 0.59 (0.57-0.62) | ＜0.001 | ＜0.001 |  | 0.61 (0.59-0.63) | ＜0.001 | ＜0.001 |
| SBP | 0.53 (0.51-0.56) | ＜0.05 | ＜0.001 |  | 0.54 (0.51-0.56) | ＜0.01 | ＜0.001 |
| DBP | 0.52 (0.49-0.55) | 0.13 | ＜0.001 |  | 0.55 (0.52-0.57) | ＜0.001 | ＜0.001 |
| Model 1 | 0.69(0.67-0.72) | ＜0.001 |  |  | 0.70(0.68-0.72) | ＜0.001 |  |
| Model 2 | 0.72(0.70-0.74)^*^ | ＜0.001 |  |  | 0.74(0.72-0.76)^*^ | ＜0.001 |  |

Data were expressed as areas under curves (95% confidence interval). Model 1 included age, gender, BMI, SBP, TG, LDL and HDL. Model 2 further included ALT/AST. ROC: receiver operating characteristics, AUC: area under the ROC curve, HOMA-IR: homeostasis model assessment-insulin resistance, ALT: alanine aminotransferase, AST: aspartate aminotransferase, TG: triglycerides, HDL: high-density lipoprotein, BMI: body mass index, WC: waist circumference, SBP: systolic blood pressure, DBP: diastolic blood pressure. *P*1: The diagnostic value for ROC, two-tailed significance. *P*2: Diﬀerence of AUCs compared to the ALT/AST ratio model, two-tailed significance (Z test). ^*^: *P*＜0.001, difference of AUCs between Model 1 and Model 2, two-tailed significance (Z test).

**Table S2 Linear regression analysis for the correlation of ALT/AST ratio and other potential metabolic factors with HOMA-IR**

|  | BMI＜25 | | | | BMI≥25 | | | |
| --- | --- | --- | --- | --- | --- | --- | --- | --- |
|  | Non-central obesity | | Central obesity | | Non-central obesity | | Central obesity | |
|  | B(95%CI) | β | B(95%CI) | β | B(95%CI) | β | B(95%CI) | β |
| ALT/AST | 0.56 (0.50,0.61)* | 0.29 | 0.55 (0.39,0.71)* | 0.26 | 0.57 (0.46,0.67)* | 0.32 | 0.68 (0.60,0.77)* | 0.35 |
| ALT | 0.16(0.12,0.21)* | 0.12 | 0.24(0.13,0.35)* | 0.16 | 0.19(0.12,0.26)* | 0.16 | 0.23(0.17,0.28)* | 0.17 |
| AST | -0.26(-0.32,-0.19)* | -0.12 | -0.03(-0.21,0.15) | -0.01 | -0.13(-0.24,-0.02)# | -0.07 | -0.10(-0.18,-0.01)# | -0.05 |
| TG | 0.27(0.23,0.31)* | 0.21 | 0.36(0.25,0.46)* | 0.25 | 0.18(0.12,0.25)* | 0.16 | 0.24(0.18,0.29)* | 0.19 |
| HDL | -0.66(-0.75,-0.57)* | -0.22 | -0.48(-0.73,-0.22)* | -0.14 | -0.30(-0.47,-0.12)& | -0.10 | -0.52(-0.66,-0.38)* | -0.16 |
| BMI | 1.47(1.27,1.67)* | 0.21 | 1.00(0.31,1.68)& | 0.11 | 0.39(-0.19,0.97) | 0.04 | 1.43(1.10,1.76)* | 0.18 |
| WC | 1.78(1.55,2.01)* | 0.26 | -0.04(-1.11,1.02) | -0.004 | 1.68(0.98,2.37)* | 0.20 | 1.74(1.32,2.16)* | 0.20 |
| SBP | 0.38(0.25,0.52)* | 0.10 | 0.33(-0.02,0.69) | 0.08 | 0.15(-0.11,0.41) | 0.04 | 0.23(0.03,0.43)# | 0.05 |
| DBP | 0.25(0.13,0.37)* | 0.06 | 0.16(0.16,0.48) | 0.04 | 0.13(-0.11,0.37) | 0.03 | 0.13(-0.06,0.32) | 0.03 |

All these indices were Ln transformed for analysis. HOMA-IR: homeostasis model assessment-insulin resistance, ALT: alanine aminotransferase, AST: aspartate aminotransferase, TG: triglycerides, HDL: high-density lipoprotein, BMI: body mass index, WC: waist circumference, SBP: systolic blood pressure, DBP: diastolic blood pressure. βvalues stand for each 1-SD increment of each potential risk factor associated with insulin resistance. The model has been adjusted for age, gender, smoking, drinking, residence area, economic status and HbA1c. #*P*＜0.05, &*P*＜0.01, **P*＜0.001.

**Table S3 Associations of insulin resistance (HOMA-IR >2) with ALT/AST ratio and other potential metabolic factors in logistic regression analyses**

|  | BMI＜25 | |  | BMI≥25 | |
| --- | --- | --- | --- | --- | --- |
|  | Non-central obesity | Central obesity |  | Non-central obesity | Central obesity |
| ALT/AST | 1.79(1.57-2.05)*** | 1.82(1.41-2.36)*** |  | 1.72(1.41-2.08)*** | 2.00(1.77-2.27)*** |
| ALT | 1.42(1.26-1.61)*** | 1.63(1.28-2.07)*** |  | 1.28(1.09-1.51)** | 1.60(1.44-1.77)*** |
| AST | 0.96(0.83-1.10) | 1.19(0.94-1.57) |  | 0.92(0.78-1.10) | 1.15(1.05-1.26)** |
| TG | 1.61(1.43-1.81)*** | 1.88(1.48-2.39)*** |  | 1.52(1.29-1.79)*** | 1.53(1.38-1.70)*** |
| HDL | 0.64(0.56-0.72)*** | 0.71(0.56-0.90)** |  | 0.72(0.60-0.87)** | 0.69(0.62-0.77)*** |
| BMI | 1.93(1.56-2.39)*** | 1.62(1.01-2.60)* |  | 1.14(0.79-1.65) | 2.13(1.79-2.52)*** |
| WC | 2.26(1.80-2.84)*** | 1.34(0.77-2.34) |  | 1.91(1.17-3.11)* | 2.40(1.96-2.93)*** |
| SBP | 1.27(1.10-1.47)** | 1.39(1.08-1.80)* |  | 1.14(0.93-1.39) | 1.16(1.04-1.30)** |
| DBP | 1.07(0.94-1.22) | 1.15(0.91-1.44) |  | 1.08(0.89-1.30) | 1.15(1.03-1.28)* |

Data were odds ratio (95% confidence interval). HOMA-IR: homeostasis model assessment-insulin resistance, ALT: alanine aminotransferase, AST: aspartate aminotransferase, TG: triglycerides, HDL: high-density lipoprotein, BMI: body mass index, WC: waist circumference, SBP: systolic blood pressure, DBP: diastolic blood pressure.

Adjusted odds ratios for each 1-SD increment of each potential risk factor associated with insulin resistance were calculated. The model has been adjusted for age, gender, smoking, drinking, residence area, economic status and HbA1c. **P*＜0.05, ***P*＜0.01, ****P*＜0.001.
